# Cardio-ankle vascular index is more closely associated than brachial-ankle pulse wave velocity with arterial damage and risk of cardiovascular disease in patients with diabetes

**DOI:** 10.1186/s12872-022-02800-9

**Published:** 2022-08-09

**Authors:** Taro Saigusa, Kentaro Watanabe, Yurika Hada, Kota Ishii, Wataru Kameda, Shinji Susa, Kenichi Ishizawa, Hisamitsu Ishihara

**Affiliations:** 1 Division of Diabetes and Metabolic Diseases, Department of Internal Medicine, Nihon University School of Medicine, 30–1 Oyaguchikami-cho, Itabashi-ku, 173-8610 Tokyo, Japan; 2 Department of Neurology, Hematology, Metabolism, Endocrinology and Diabetology, Yamagata University Faculty of Medicine, 2-2-2 Iida-nishi, 990-9585 Yamagata, Japan

**Keywords:** Cardio-ankle vascular index, Brachial-ankle pulse wave velocity, Arterial damage, Risk of cardiovascular disease

## Abstract

**Background:**

This study aimed to compare the usefulness of arterial stiffness parameters, cardio-ankle vascular index (CAVI) and brachial-ankle pulse wave velocity (baPWV), for evaluating arterial damage and risk of cardiovascular disease (CVD) in subjects with diabetes.

**Methods:**

The study subjects were 277 patients with type 1 or type 2 diabetes. All subjects were evaluated for vascular stiffness using CAVI (n = 154) or baPWV (n = 123). Carotid intima-media thickness (IMT) and the Suita score were also measured because these are established risk factors for future CVD. Associations of both CAVI and baPWV with these established parameters were evaluated in all subjects, and then in 174 subjects with adjustment for covariates by using propensity score matching.

**Results:**

In all subjects, CAVI and baPWV correlated significantly with both IMT (r = 0.462, *P* < 0.001, and r = 0.212, *P* = 0.019, respectively) and the Suita score (r = 0.573, *P* < 0.001, and r = 0.373, *P* < 0.001, respectively). The correlation between CAVI and IMT was more significant than that between baPWV and IMT (Z = 2.33, *P* = 0.020). Similarly, the correlation between CAVI and the Suita score was more significant than that between baPWV and the Suita score (Z = 2.13, *P* = 0.033). After adjustment by propensity score matching, significant correlations between CAVI and IMT (r = 0.432 *P* < 0.001) and between CAVI and the Suita score (r = 0.544, *P* < 0.001) were preserved, though only the association between baPWV and the Suita score was significant (r = 0.289, *P* = 0.007) while that between baPWV and IMT showed no significance. Again, CAVI showed a significant association with the Suita score than baPWV (Z = 2.02, *P* = 0.043).

**Conclusions:**

CAVI is more closely associated than baPWV with arterial damage and risk of CVD in patients with diabetes.

**Supplementary Information:**

The online version contains supplementary material available at 10.1186/s12872-022-02800-9.

## Backgrounds

Subjects with dyslipidemia, hypertension and diabetes mellitus, who smoke, have a high risk of developing cardiovascular disease (CVD) [1]. Furthermore, CVD risk factor clusters reportedly raised CVD risk in a general population cohort [2]. Therefore, evaluating the risk of developing CVD is important for improving CVD mortality in patients with diabetes who have CVD risk factors. Among many tools for CVD risk assessment, brachial-ankle pulse wave velocity (baPWV) [3] and cardio-ankle vascular index (CAVI) [4] are useful and noninvasive. Both baPWV and CAVI evaluate vascular stiffness, which is recognized as a surrogate marker predicting CVD risk [5–8]. It is still debated whether either CAVI or baPWV is useful for evaluating atherosclerosis and CVD risk [9–11]. In Japan, CAVI and baPWV measurements are widely performed and used for evaluating arterial stiffness because these variables can be measured easily. Since most of physicians in Japan recognize that there is little difference in significance between CAVI and baPWV as evaluation variable of vascular stiffness, hospitals or clinics choose either equipment of CAVI or baPWV measurement in daily practice. In our knowledge, it has not been shown whether CAVI or baPWV measurement is a better tool for detecting arterial damage in patients with diabetes.

Thus, we aimed to compare usefulness for cardiovascular risk assessment between baPWV and CAVI in patients with diabetes. We selected carotid intima-media thickness (IMT) and the Suita score for evaluating the arterial damage and the risk of CVD development, respectively. Carotid IMT reflects arterial damage that is induced by accumulation of past exposure to CVD risk factors [12] and widely recognized as a surrogate marker for the risk of CVD in the future [13, 14]. Additionally, the Suita score provides suitable risk factor categories for predicting the ten-year probability of coronary heart disease (CHD), and is more accurate for predicting CHD risk than the Framingham risk score in the Japanese population [15].

## Methods

### Study subjects

Two hundred seventy-seven patients with type 1 or type 2 diabetes (173 men and 104 women including 9 type 1 diabetes patients, average age 64.8 ± 11.5 years) were recruited as study subjects. All subjects were ambulatory and were followed at the Department of Neurology, Hematology, Metabolism, Endocrinology and Diabetology, Yamagata University Faculty of Medicine and Division of Diabetes and Metabolic Diseases, Department of Internal Medicine, Nihon University School of Medicine. Patients with atrial fibrillation, peripheral arterial disease, malignant diseases, collagen diseases, acute and chronic inflammatory diseases, and/or receiving steroid hormone therapy or other immunosuppressants, were excluded from this study.

### Characteristics of study subjects

We determined clinical characteristics including sex, age, body mass index (BMI), smoking habit, systolic and diastolic blood pressures, anti-hypertensive drug use, statin use, and biochemical variables in all subjects. Biochemical variables, including lipid metabolic parameters, uric acid and HbA1c were measured after an overnight fast. Low-density lipoprotein (LDL) cholesterol, high-density lipoprotein (HDL) cholesterol, triglycerides, uric acid, creatinine and HbA1c were measured using an automatic analyzer. The estimated glomerular filtration rate (eGFR) served as an indicator of renal function. eGFR was estimated by the following formula: eGFR (mL/min/1.73 m^2^) = 194 × Serum creatinine^−1.094^ × Age^−0.287^ × 0.739 (if female) [16]. Blood pressure was measured with the patient in a sitting position at the hospital in the morning.

### Examination of atherosclerosis and cardiovascular risk

Carotid IMT, CAVI and baPWV were measured as variables associated with atherosclerosis. Carotid IMT was established as a suitable surrogate marker for the risk of future CVD development [13, 14]. A total of six segments of the near and far walls in the common carotid artery, at the bifurcation, and in the internal carotid artery on the right and left were measured with B‐mode imaging of ultrasonography, as described in a previous report [17]. The maximum IMT, including bilateral plaque, was defined as the IMT in all study subjects [17]. Previous studies indicated that maximum IMT reflects well to target organ damage [18] or risk of CVD in Japanese cohort [19]. Carotid ultrasonographic measurements were performed by experienced clinician. The IMT measurements showed a variability of 8.0%, as previously reported [17].

CAVI [4] and baPWV [3] are indicators of arterial stiffness. CAVI is an index of arterial stiffness based on the stiffness parameter β [4], while baPWV reflects the stiffness from the aorta to the lower limb arteries [3]. CAVI [5, 6] and baPWV [7, 8] have been recommended to surrogate markers for CVD. CAVI was measured using a Vasera VS-1000 vascular screening system (Fukuda Denshi, Tokyo, Japan). The maximum CAVI on both sides was recorded in each of the study subjects (n = 154) enrolled at Yamagata University Hospital and the maximum baPWV measurement was performed using a form PWV/ABI (Omron Healthcare Co., Ltd. Kyoto, Japan) in subjects (n = 123) enrolled at Nihon University Itabashi Hospital. CAVI and baPWV in this study were defined as the largest CAVI and baPWV between those of both sides.

**Fig. 1:**
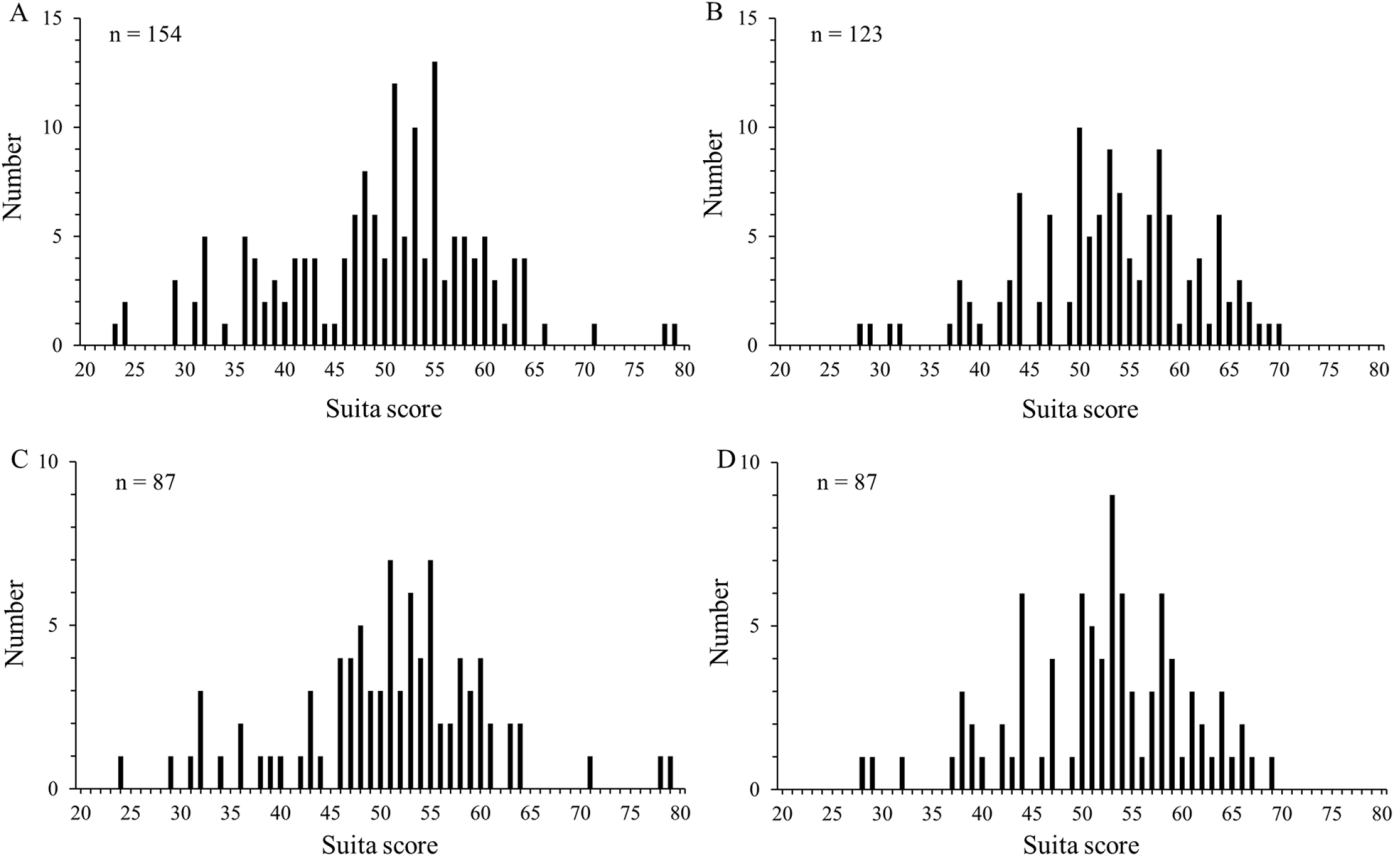
The distributions of Suita score in subjects before and after adjustment by propensity score matching. **A** subjects measured CAVI before adjustment by propensity score matching, **B** subjects measured baPWV before adjustment by propensity score matching, **C** subjects measured CAVI after adjustment by propensity score matching, **D** subjects measured baPWV after adjustment by propensity score matching. CAVI; cardio-ankle vascular index, baPWV; brachial-ankle pulse wave velocity

For predicting the risk of CVD development, we used the Suita score. The Suita score is an established cardiovascular risk score based on risk factor categories for predicting CHD in the Japanese population [15]. The Suita score consists of the sum of each of these four risk categories and indicates the ten-year probability of CHD [15]. The components of Suita score and prediction score sheet was indicated in Additional file 2: Table S1. The distribution of Suita score in subjects before and after adjustment by propensity score matching were shown in Fig. 1.

### Statistical analysis

All subjects were divided into the CAVI (n = 154) and baPWV groups (n = 123). Furthermore, to adjust for clinical characteristics, we performed one-to-one propensity score matching [20]. Each patient in the CAVI group was paired with a patient in the baPWV group based on the propensity score in this one-to-one matching. Propensity scores were calculated using logistic regression analysis with the covariates of sex, age, BMI, smoking, systolic and diastolic blood pressure, receiving statins and antihypertensive drugs, HDL cholesterol, LDL cholesterol, triglyceride, uric acid, eGFR and HbA1c. Patients with the nearest propensity score within the caliper were paired. A caliper size in the range of 0.20 to 0.25 standard deviation (SD) is recommended [21, 22]. This study defined a caliper as 0.20 SD. As a result, 87 patients were in both the CAVI and the baPWV group after propensity score matching. The Mann–Whitney U test and the chi-square test were performed to compare clinical characteristics between the CAVI and baPWV groups before or after adjustment by propensity score matching. Bonferroni’s multiple comparison test was used to compare the mean values of CAVI and baPWV between age groups and IMT between groups in combination high or low CAVI or baPWV groups with high or low the Suita score or eGFR after adjustment by propensity score matching method. Spearman's correlation coefficient and univariate linear regression analysis were used to identify whether CAVI and the Suita score were significantly associated with clinical characteristics, IMT and the Suita score in subjects before or after adjustment by propensity score matching. In this univariate linear regression analysis, we assumed CAVI and baPWV to be dependent variables, and sex (men), smoking habit (current), anti-hypertensive drug use or statin use to be independent variables. The observed Z test was used to compare and analyze statistical significance between correlation coefficients. The difference in variance between CAVI and baPWV was evaluated using the F-test of equality of variances. Data are presented as means ± SD, number (%), coefficient of covariation (r), Z values (z), F-value or β coefficients. A value of *P* < 0.05 was considered to indicate statistical significance. All analyses were performed with IBM SPSS Statistics for Windows Version 25 J (IBM Corp., Armonk, NY, USA).

## Results

### Clinical characteristics of study subjects

The characteristics of the CAVI and baPWV groups before and after propensity score matching are shown in Table 1. Sex (*P* = 0.041), age (*P* = 0.002), systolic blood pressure (*P* = 0.007), statin use (*P* < 0.001), LDL cholesterol (*P* = 0.020), eGFR (*P* < 0.001) and HbA1c (*P* < 0.001) differed significantly between the CAVI and baPWV groups before adjustment by propensity score matching (Table 1).

**Table 1:** Clinical characteristics of study subjects

Clinical characteristics	Before adjustment by propensity score matching		After adjustment by propensity score matching	
	CAVI group	baPWV group	CAVI group	baPWV group
	(n = 154)	(n = 123)	(n = 87)	(n = 87)
Sex (men)	88 (57.1)	85 (69.1)^#^	52 (59.8)	53 (60.9)
Age (years)	62.9 ± 12.3	67.0 ± 10.0"	65.1 ± 12.3	67.0 ± 10.9
Body mass index	24.60 ± 4.89	24.71 ± 3.85	24.59 ± 4.76	24.77 ± 3.91
Smoking habit (current)	34 (22.1)	30 (24.4)	22 (25.3)	16(18.4)
Blood pressure (nrmHg)				
Systolic	127.0 ± 16.5	133.0 ± 18.2"	130.3 ± 15.4	132.3 ± 19.1
Diastolic	76.3 ± 10.9	77.0 ± 11.7	76.4 ± 11.2	77.1 ± 11.9
Antihypertensive drug use	93 (60.4)	84 (68.3)	59 (67.8)	56 (64.4)
Statin use	69 (44.8)	81 (65.9)**	44 (50.6)	52 (59.8)
HDL-CHOL (mmol/L)	1.42 ± 0.53	1.42 ± 0.37	1.48 ± 0.53	1.45 ± 0.41
LDL-CHOL (mmol/L)	2.77 ± 0.80	2.57 ± 0.78*	2.63 ± 0.69	2.68 ± 0.80
Triglyceride (mmol/L)	1.62 ± 1.02	1.42 ± 0.77	1.34 ± 0.74	1.41 ± 0.67
Uric acid (umol/L)	323.1 ± 90.1	316.6 ± 70.4	319.3 ± 81.6	316.3 ± 70.7
eGFR (mL/min/1.73 m2)	83.49 ± 33.21	69.82 ± 17.98**	77.08 ± 27.46	70.64 ± 19.65
HbAlc (%)	9.01 ± 2.41	7.14 ± 0.84**	7.66 ± 1.43	7.26 ± 0.89

Mean ± SD, n (%)

^*^P < 0.05, ^**^P < 0.01 vs CAVI group by Mann–Whitney U test; ^#^*P* < 0.05 vs CAVI group by Chi-squire test

HDL, high-density lipoprotein; LDL, low-density lipoprotein; eGFR, estimated glomerular filtration rate; HbA1c, glycosylated hemoglobin

### Associations between CAVI or baPWV and clinical characteristics

Mean values of CAVI and baPWV in all study subjects were 8.69 and 17.74 m/sec., respectively. Age showed significant associations between CAVI and baPWV both before (r = 0.653 and 0.550, respectively, *P* < 0.001) and after (r = 0.617 and 0.551, respectively, *P* < 0.001) adjustment by propensity score matching (Table 2). Additionally, variations in baPWV in each age group or all subjects were significantly greater those in CAVI (Additional file 2: Table S2). Adjustment by propensity score matching did not change the influence of age on CAVI and baPWV (data not shown).

**Table 2:** Correlation coefficient between CAVI, baPWV and clinical characteristics

Clinical characteristics	Before adjustment by propensity score matching		After adjustment by propensity score matching	
	CAVI group	baPWV group	CAVI group	baPWV group
	(n = 154)	(n = 123)	(n = 87)	(n = 87)
Age (years)	0.653**	0.550**	0.617**	0.551**
Body mass index	− 0.291**	− 0.220*	− 0.427**	− 0.216*
Blood pressure (mmHg)				
Systolic	0.325**	0.337**	0.379**	0.377**
Diastolic	0.107	0.014	0.154	0.061
HDL-CHOL (mmol/L)	0.094	− 0.008	0.163	0.035
LDL-CHOL (mmol/L)	− 0.095	− 0.131	0.0008	− 0.238*
Triglyceride (mmol/L)	− 0.227**	− 0.177*	− 0.197	− 0.218*
Uric acid (umol/L)	− 0.062	− 0.105	− 0.181	− 0.205
eGFR (mL/min/1.73 m^2^)	− 0.255*	− 0.190*	− 0.293^**^	− 0.134
HbAlc (%)	− 0.143	0.029	− 0.017	0.076

^*^P < 0.05, ^**^P < 0.01

HDL, high-density lipoprotein; LDL, low-density lipoprotein; eGFR, estimated glomerular filtration rate; HbA1c, glycosylated hemoglobin

CAVI showed significant associations with BMI (r = − 0.291, *P* < 0.001), systolic blood pressure (r = 0.325, *P* < 0.001), Triglyceride (r = − 0.227, *P* = 0.005) and eGFR (r = − 0.255, *P* = 0.001) before adjustment by propensity score matching (Table 2). After adjustment, associations of CAVI with BMI (r = − 0.427, *P* < 0.001), systolic blood pressure (r = 0.379, *P* < 0.001) and eGFR (r = − 0.293, *P* = 0.006) remained significant (Table 2).

baPWV showed significant associations with BMI (r = − 0.220, *P* = 0.014), systolic blood pressure (r = 0.337, *P* < 0.001), triglyceride (r = − 0.177, *P* = 0.049) and eGFR (r = − 0.190, *P* = 0.036) before adjustment by propensity score matching (Table 2). After adjustment by propensity score matching, the associations remained significant for BMI (− 0.216, *P* = 0.045), systolic blood pressure (r = 0.377, *P* < 0.001) and triglyceride (r = − 0.218, *P* = 0.043), and LDL cholesterol was also found to show a significant association with baPWV (r = − 0.238, *P* = 0.026) (Table 2).

Univariate linear regression analysis indicated significant associations between baPWV and antihypertensive drug use (β = 1.547, *P* = 0.029) before adjustment by propensity score matching (Table 3). After adjustment by propensity sore matching, however, current smoking was found to show a significant correlation (β = − 1.195, *P* = 0.026), while the significant association with antihypertensive drug use was unchanged (β = 1.796, *P* = 0.031) (Table 3).

**Table 3:** Univariate linear regression analysis predicting for association between clinical characteristics, CAVI and baPWV

Clinical characteristics	Before adjustment by propensity score matching		After adjustment by propensity score matching	
	CAVI group	baPWV group	CAVI group	baPWV group
	(n = 154)	(n = 123)	(n = 87)	(n = 87)
Sex (men)	0.291 (− 0.173–0.756)	0.059 (− 1.363–1.480)	0.262 (− 0.353–0.876)	− 0.102 (− 1.744–1.539)
Smoking habit (current)	− 0.076 (− 0.372–0.220)	− 0.420 (− 1.264–0.424)	− 0.104 (− 0.484–0.276)	− 1.195 (− 2.243−0.147)*
Antihypertensive drug use	0.408 (− 0.059–0.876)	1.547 (0.163–2.931)*	0.281 (− 0.363—0.926)	1.796 (0.168–3.423)*
Statin use	0.309 (− 0.153–0.770)	0.645 (− 0.735–2.026)	− 0.207 (0.810–0.397)	0.792 (− 0.832–2.417)

*P < 0.05

Dependent variable: CAVI or baPWV, independent variables: sex (men), smoking habit (current), antihypertensive drug use or statin use

CAVI, cardio-ankle vascular index; baPWV, brachial-ankle pulse wave velocity

### Associations of CAVI and baPWV with cardiovascular risk factors

**Fig. 2:**
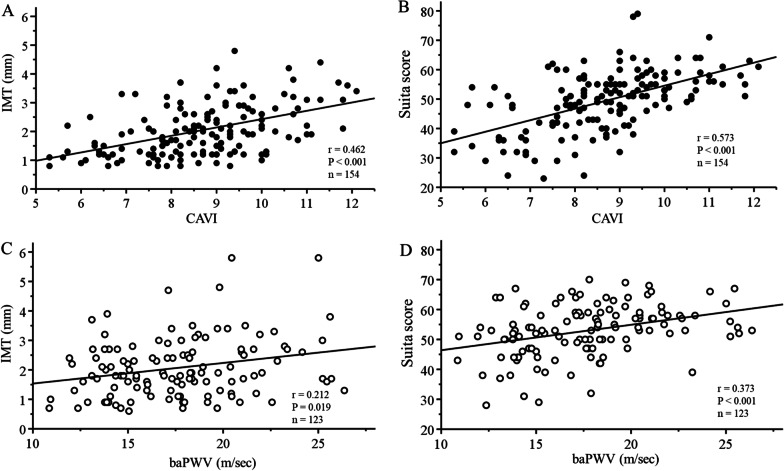
Correlations of CAVI and baPWV with IMT and the Suita score before adjustment by propensity score matching. **A** CAVI and IMT, **B** CAVI and Suita score, **C** baPWV and IMT, **D** baPWV and Suita score. CAVI; cardio-ankle vascular index, baPWV; brachial-ankle pulse wave velocity, IMT; intima-media thickness

CAVI and baPWV showed significant correlations with both IMT (r = 0.462, *P* < 0.001, and r = 0.212, *P* = 0.019, respectively) (Fig. 2A, 2C) and the Suita score (r = 0.573, *P* < 0.001, and r = 0.373, *P* < 0.001, respectively) (Fig. 2B, 2D). The correlations between CAVI and IMT and the Suita score were both more significant than those between baPWV and IMT (Z = 2.33, *P* = 0.020) and the Suita score (Z = 2.13, *P* = 0.033).

**Fig. 3:**
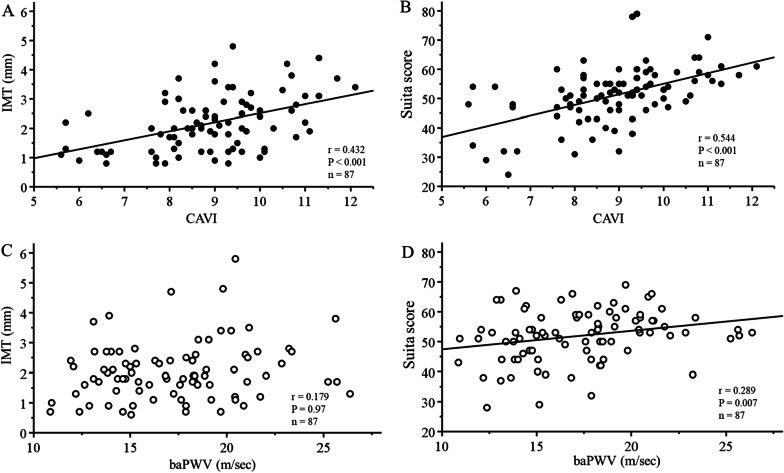
Correlations of CAVI and baPWV with IMT and the Suita score after adjustment by propensity score matching. **A** CAVI and IMT, **B** CAVI and Suita score, **C** baPWV and IMT, D: baPWV and Suita score. CAVI; cardio-ankle vascular index, baPWV; brachial-ankle pulse wave velocity, IMT; intima-media thickness

After adjustment by propensity score matching, the significant correlations of CAVI with IMT (r = 0.432, *P* < 0.001) (Fig. 3A) and the Suita score (r = 0.544, *P* < 0.001) (Fig. 3B) were unchanged. Although baPWV showed no significant association with IMT (Fig. 3C), baPWV was only significantly associated with the Suita score (r = 0.289, *P* = 0.007) (Fig. 3D). Again, CAVI showed a significantly stronger association with the Suita score than did baPWV (Z = 2.02, *P* = 0.043).

### Predictive ability for higher value of IMT in combination CAVI or baPWV with the Suita score or eGFR after adjustment by propensity score matching

**Fig. 4:**
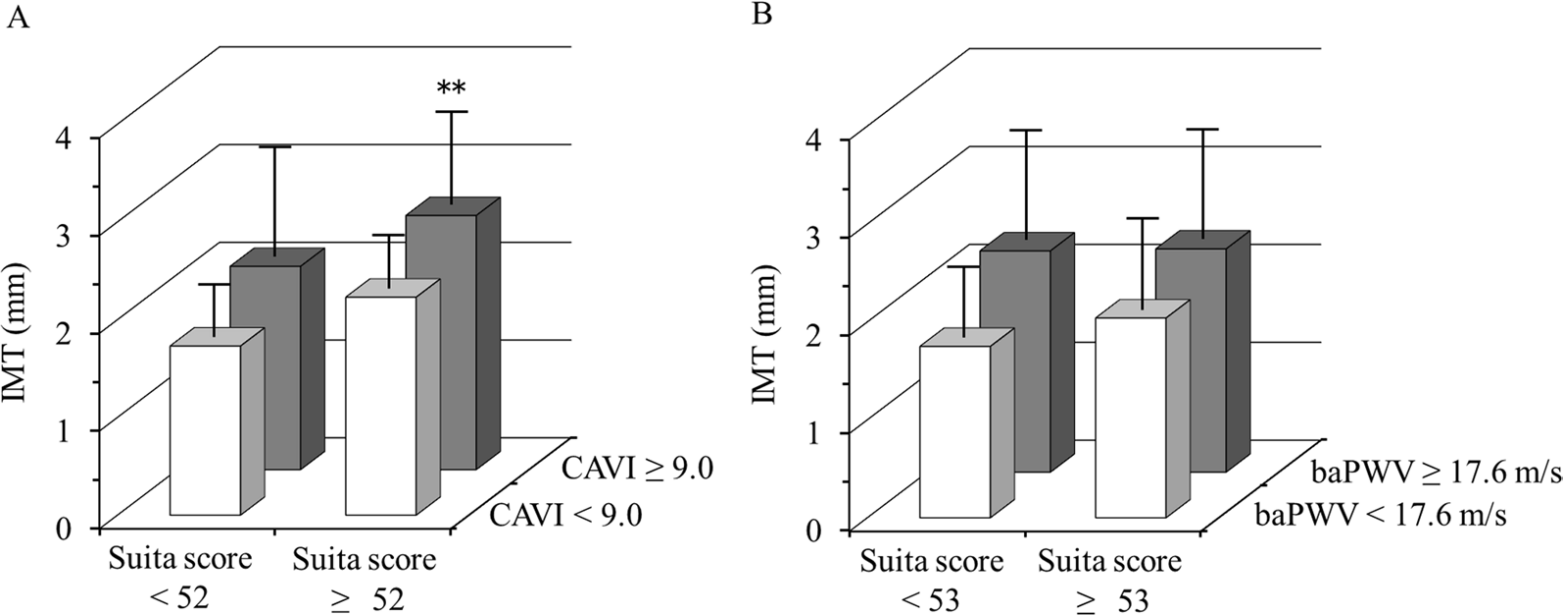
Mean IMT of groups in combination high or low CAVI or baPWV groups with high or low the Suita score after adjustment by propensity score matching. **A** combination CAVI with the Suita score, **B** combination baPWV with the Suita score. CAVI; cardio-ankle vascular index, baPWV; brachial-ankle pulse wave velocity, IMT; intima-media thickness. **P < 0.01 vs group with low CAVI and low Suita score

Subjects were categorized as groups with low or high CAVI, baPWW, Suita score or eGFR based on cutoff values (median of each variable) after adjustment by propensity score matching. The predictive ability for the higher value of IMT in combination CAVI or baPWV with the Suita score or eGFR was evaluated using these groups. The additive value of CAVI to the Suita score for predicting a higher value of IMT was improved, while combination of baPWV with the Suita score could not improve the predictive power of IMT after adjustment by propensity score matching. The mean IMI in subjects with high CAVI (≥ 9.0) and high Suita score (≥ 52) was significantly higher than that in subjects with low CAVI and low Suita score (2.60 ± 0.93 mm and 1.73 ± 0.71, respectively, *P* = 0.002) (Fig. 4A). Conversely, combination of baPWV with the Suita score could not contribute to improve predictive power of IMT (Fig. 4B).

Additionally, the predictive ability of IMT in combination CAVI or baPWV with eGFR showed the same results as with the Suita score (Additional file 1: Supplemental Figure).

## Discussion

Our results indicate both CAVI and baPWV to be significantly associated with carotid IMT and the Suita score, widely used surrogate variables for assessing atherosclerosis and the risk of developing CVD in the future. Notably, CAVI was found to be more closely associated than baPWV with arterial damage and risk of CVD in patients with diabetes. The significance of examination about the correlation between CAVI or baPWV and Suita score or carotid IMT is that these correlations provide useful information about whether CAVI or baPWV is the more suitable arterial stiffness variable to assess the past exposure to the risk of cardiovascular disease (CVD) or the severity of atherosclerosis risk. To our knowledge, this is the first study to demonstrate a difference in clinical significance between CAVI and baPWV in patients with diabetes. The notable point of our study is that we could indicated a statistical assessment of which of CAVI and baPWV is superior in evaluating arterial damage and CVD risk in patients with diabetes. CAVI kept the significant associations with both IMT and Suita score before and after adjustment by propensity score matching, although baPWV only indicated significant associations with IMT and Suita score before adjustment by propensity score matching. Further, we have statistically proven using observed Z test that CAVI was more closely correlated than baPWV with both IMT and Suita score before and after adjustment by propensity score matching. Additionally, we have indicated that combination CAVI with the Suita score more improve than baPWV in predictive value of arterial damage. The strength of our study is that we indicated CAVI is a suitable tool for evaluating arterial damage and risk of CVD accurately compared to baPWV in patients with diabetes, although CAVI and baPWV show significant associations with traditional CVD risk factors. Therefore, we concluded that CAVI is a more appropriate tool than baPWV to compare the risk of CVD between individuals with diabetes.

We can speculate as to why CAVI more closely reflects atherosclerosis and the risk score of CVD than baPWV in patients with diabetes. One possibility is that peripheral arterial stiffness influences CAVI and baPWV measurements. CAVI reflects central arterial stiffness because the basic formula for calculating CAVI is based on heart-ankle PWV [4]. CAVI is more influenced by central arterial stiffness than baPWV because the route of the pulse wave in baPWV reflects the status of both central and peripheral arteries, including those from the aortic annulus to the brachium [23]. Atherosclerosis, renal and cardiac function are reportedly more closely associated with central arterial stiffness (carotid-femoral PWV) than baPWV [24]. Furthermore, a past study of patients with chest pain syndrome showed that carotid IMT had a somewhat more significant association with CAVI than with baPWV [9].

Another factor is that CAVI shows higher reproducibility than baPWV. We found variations in baPWV in each age group as well as in all subjects to be significantly larger than those in CAVI. We thus suggest that the blood pressure at the time of measurement may influence the difference in variation between CAVI and baPWV. This study demonstrated significant associations of systolic blood pressure with both CAVI and baPWV. baPWV is significantly influenced by antihypertensive medication [25] or variation of blood pressure [26], but CAVI does not change [25]. Further, baPWV depends on blood pressure at the time of measurement [25]. In contrast, CAVI was reported to be significantly, but more weakly than baPWV, correlated with blood pressure [26]. The blood pressure variability in patients with diabetes is high compared to that in subjects with normal glucose tolerance [4]. In addition, this variability increases with age [27]. Further, the accuracy of the path length formula also influences the variation in both CAVI and baPWV measurements. Magnetic resonance imaging results established that the estimated path length from the heart to the ankle in CAVI well reflects the true path length [28]. Conversely, it is proven that the estimated path length from the aortic annulus to the brachium in the baPWV measurement is shorter than the actual path length [23].

As prior studies documented, our study also indicated significant associations between traditional CVD risk factors and both CAVI and baPWV. Our study showed age-related increases in CAVI and baPWV. The elevations of CAVI and baPWV with age have previously been documented [6, 29] because increasing aortic stiffness with age contributes to the observed increases in CAVI [30] and baPWV [31]. Interestingly, we demonstrated CAVI and baPWV to be inversely associated with BMI. Previous cross-sectional studies indicated CAVI and baPWV to correlate negatively with BMI [32, 33]. We speculate that BMI is calculated as body weight/height squared, and BMI would thus be expected to have an inverse association with CAVI and baPWV.

Additionally, the invert associations between baPWV and triglyceride, LDL cholesterol or smoking habit were indicated in our study. The reasons for these inverse associations have yet to be clarified. BMI was positively associated with triglyceride (data not shown) in our study. It is reasonable to speculate that the positive association between BMI and triglyceride in our study explains the significant inverse association between baPWV and triglyceride. The significant positive association between LDL cholesterol and triglyceride (data not shown) also may explain the invert association between baPWV and LDL cholesterol. Subjects with smoking habit were younger, and indicated higher prevalence of the use of antihypertensive medication than subjects without (data not shown). We concluded that receiving antihypertensive medication and the age in subjects with smoking habit may cause the invert association between baPWV and smoking habit.

There were several possible limitations in our study. First, we could not perform CAVI and baPWV measurement at the same time in one population. Confirmed evidences are lacking as to whether CAVI or baPWV is more suitable for evaluating arterial damage in diabetes patients, and physicians usually consider no differences between the two. In addition, hospitals or clinics choose either CAVI or baPWV equipment for financial reasons. Hence, we have no data of CAVI and baPWV measured in one population and used data from two populations. In order to minimize background difference, we have conducted the propensity matching method in the present study. Indeed, past study that compared the significances of two indices using different cohort and propensity score matching method was present [34]. Additionally, the number of our study subjects was relatively small to compare the significance using the propensity score matching method. However, the previous study that compared the outcome between video-associated thoracoscopic surgery lobectomy (n = 64) and stereotactic ablative radiotherapy (n = 64) for early-stage non-small cell lung cancer using propensity score-matched analysis and two different small population existed [35]. However, unadjusted confounding factors we did not expected in two different populations may affect the results of our study because we selected pairs of subjects using backgrounds mainly associated with arterial damage or CVD risk. Hence, comparing the two indices in different populations might be inappropriate. Whether CAVI is superior to baPWV should be confirmed in one cohort in future. Second, we could not evaluate the difference in correlations between mean IMT and both CAVI and baPWV because mean IMT was measured only subjects with CAVI measurements. Mean IMT also showed significant correlation with CAVI (r = 0.368, *P* < 0.001) before adjustment by propensity score matching method. Mean IMT reflects organ damage [36]. It is necessary to examine the correlations between mean IMT and both CAVI and baPWV to strengthen the evidence of our study. Third, we could not provide the difference in associations between the duration of diabetes or hypoglycemic medication and CAVI or baPWV. We only have a dataset of the duration of diabetes in subjects receiving CAVI measurement and almost all subjects received several hypoglycemic medications. Subjects with high CAVI [37] or baPWV [38] showed a long duration of diabetes. CAVI showed a significant positive correlation with the duration of diabetes (r = 0.309, *P* < 0.001) before propensity score matching method. Past prospective studies have reported that medications of diabetes improved CAVI [39] or baPWV [40] independent of HbA1c. It is difficult to evaluate the effect of medication of diabetes on CAVI or baPWV in cross-sectional analysis. Fourth, we indicated our study results using CAVI and baPWV that are measured once only. The reproducibility and accuracy of our study results have to be evaluated using CAVI and baPWV that are measured at the different times. Fifth, the difference in predictive power in the risk of CVD development between CAVI and baPWV was not demonstrated prospectively. A prospective study is needed to prove the evidence of our study. Finally, a strong match with the Suita score may not be a requirement for CAVI or baPWV since the Suita does not reveal the severity of atherosclerosis. There is a time lag between risks of CVD development evaluated by both IMT and the Suita score because IMT is an organic stiffness parameter, whereas the Suita score is the severity of atherosclerosis risk. The finding that CAVI is a more suitable variable than baPWV to assess the high value of IMT is important to evaluate the superiority of CAVI in arterial stiffness variables to baPWV. However, we considered that the superiority of CAVI in association with the Suita score provides useful information to assess whether CAVI or baPWV is a superior variable to evaluate atherosclerosis.

## Conclusion

Our study demonstrated that CAVI is more closely associated than baPWV with arterial damage and risk of CVD in patients with diabetes. Thus, CAVI might be a more suitable tool for cardiovascular risk assessment than baPWV. CAVI appears to be particularly useful for assessing patients with diabetes who show a high risk for developing CVD.

## Supplementary Information

**Additional file 1. Supplemental figure.** Mean IMT of groups in combination high or low CAVI or baPWV groups with high or low eGFR after adjustment by propensity score matching. A: combination CAVI with eGFR, B: combination baPWV with eGFR. CAVI; cardio-ankle vascular index, baPWV; brachial-ankle pulse wave velocity, IMT; intima-media thickness, eGFR; estimated glomerular filtration rate. **P < 0.01 vs group with low CAVI and high eGFR.

**Additional file 2. Supplemental Table S1.** Prediction score sheet for Suita score. **Supplemental Table S2.** Comparison of variation in CAVI and baPWV in each age groups or all subjects.

## Data Availability

Data available on request from the correspondence author (Kentaro Watanabe; watanabe.kentaro@nihon-u.ac.jp).

## Abbreviations

CAVI: Cardio-ankle vascular index
baPWV: Brachial-ankle pulse wave velocity
CVD: Cardiovascular disease
IMT: Intima-media thickness
CHD: Coronary heart disease
BMI: Body mass index
LDL: Low-density lipoprotein
HDL: High-density lipoprotein
eGFR: Estimated glomerular filtration rate
SD: Standard deviation
