# Supplementary material for: Cardio-ankle vascular index is more closely associated than brachial-ankle pulse wave velocity with arterial damage and risk of cardiovascular disease in patients with diabetes
Source: BMC Cardiovasc Disord. 2022 Aug 9;22:365. doi: 10.1186/s12872-022-02800-9 (PMC9364514; doi:10.1186/s12872-022-02800-9)
Supplement: Supplementary file 1 — Additional file 1. Supplemental figure. Mean IMT of groups in combination high or low CAVI or baPWV groups with high or low eGFR after adjustment by propensity score matching. A: combination CAVI with eGFR, B: combination baPWV with eGFR. CAVI; cardio-ankle vascular index, baPWV; brachial-ankle pulse wave velocity, IMT; intima-media thickness, eGFR; estimated glomerular filtration rate. **P < 0.01 vs group with low CAVI and high eGFR. [file 12872_2022_2800_MOESM1_ESM.docx]

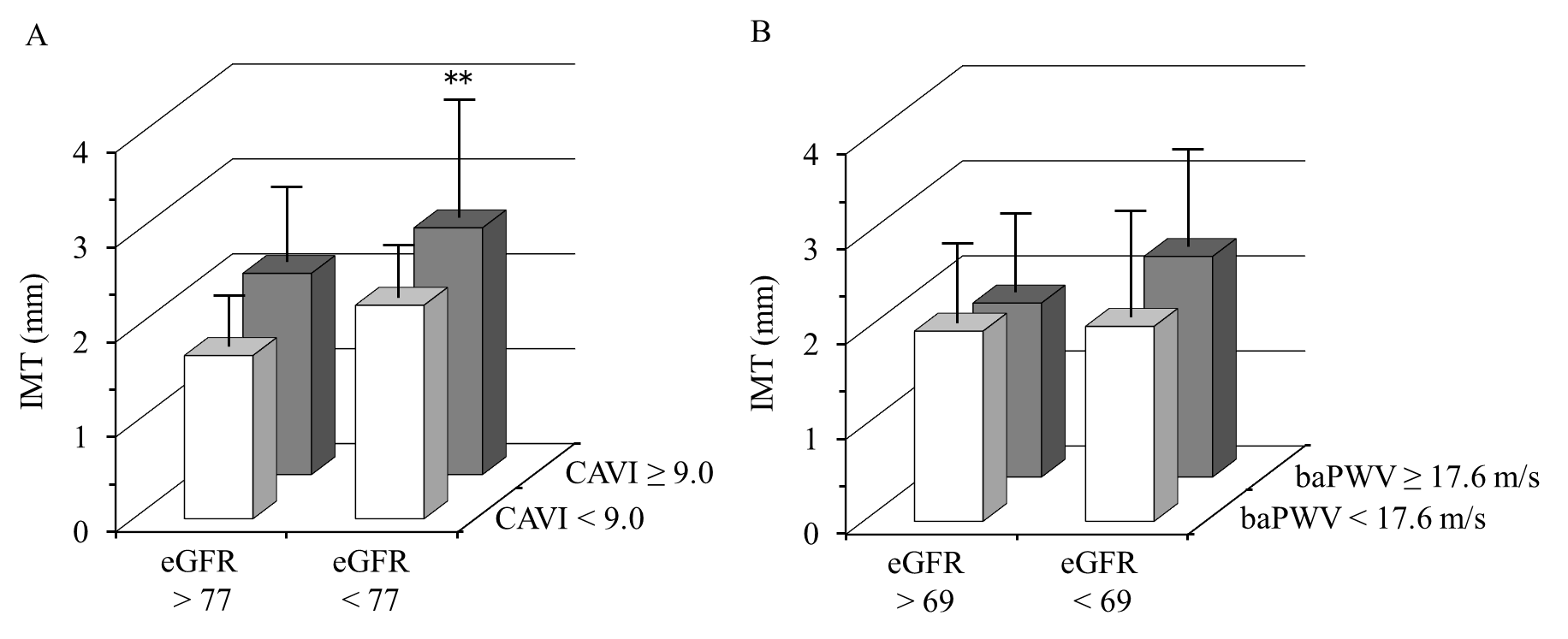


**P < 0.01 vs group with low CAVI and high eGFR.

Mean IMT of groups in combination high or low CAVI or baPWV groups with high or low eGFR after adjustment by propensity score matching. A: combination CAVI with eGFR, B: combination baPWV with eGFR. CAVI; cardio-ankle vascular index, baPWV; brachial-ankle pulse wave velocity, IMT; intima-media thickness, eGFR; estimated glomerular filtration rate.

Supplemental Figure
