# Supplementary material for: Cardio-ankle vascular index is more closely associated than brachial-ankle pulse wave velocity with arterial damage and risk of cardiovascular disease in patients with diabetes
Source: BMC Cardiovasc Disord. 2022 Aug 9;22:365. doi: 10.1186/s12872-022-02800-9 (PMC9364514; doi:10.1186/s12872-022-02800-9)
Supplement: Supplementary file 2 — Additional file 2. Supplemental Table S1. Prediction score sheet for Suita score. Supplemental Table S2. Comparison of variation in CAVI and baPWV in each age groups or all subjects. [file 12872_2022_2800_MOESM2_ESM.docx]

Supplemental Table S1 Prediction score sheet for Suita score.


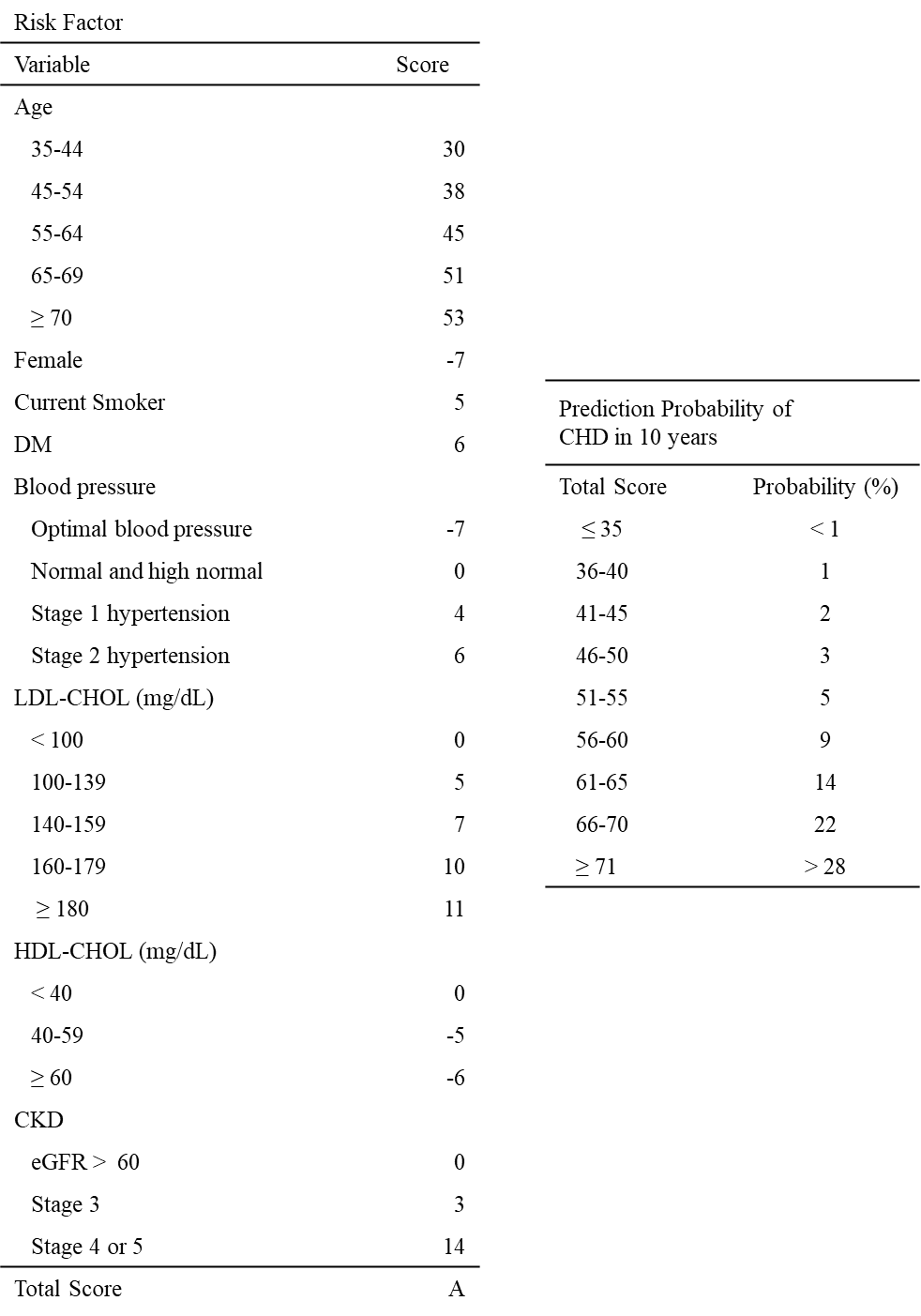


DM; diabetes, LDL-CHOL; low-density lipoprotein cholesterol, HDL-CHOL; high-density lipoprotein cholesterol, CKD; chronic kidney disease, eGFR; estimated glomerular filtration rate, CHD; coronary heart disease.

Supplemental Table S2 Comparison of variation in CAVI and baPWV in each age groups or all subjects.


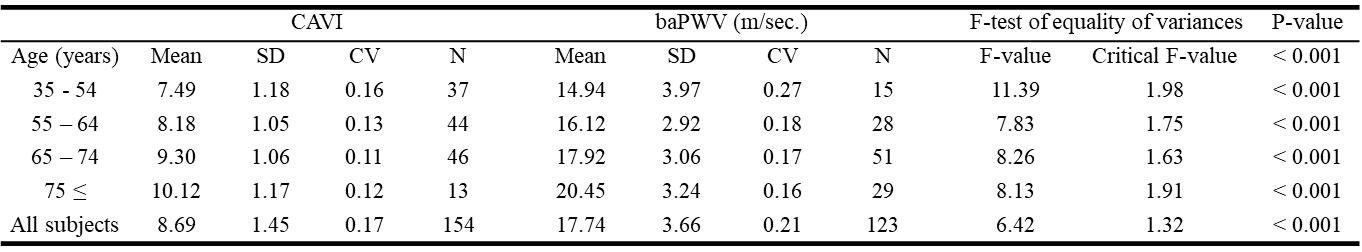


CAVI, cardio-ankle vascular index; baPWV, brachial-ankle pulse wave velocity; SD, standard deviation; CV, coefficient of variation.
